# *N*-(3,5-Dimethyl­phen­yl)benzene­sulfonamide

**DOI:** 10.1107/S1600536809050089

**Published:** 2009-11-28

**Authors:** P. G. Nirmala, B. Thimme Gowda, Sabine Foro, Hartmut Fuess

**Affiliations:** a Department of Chemistry, Mangalore University, Mangalagangotri 574 199, Mangalore, India; b Institute of Materials Science, Darmstadt University of Technology, Petersenstrasse 23, D-64287 Darmstadt, Germany

## Abstract

In the crystal structure of the title compound, C_14_H_15_NO_2_S, the mol­ecule is bent at the S atom with a C—SO_2_—NH—C torsion angle of 67.9 (2)°. The two benzene rings are tilted by 54.6 (1)° relative to each other. In the crystal, inter­molecular N—H⋯O hydrogen bonds pack the mol­ecules into a supra­molecular structure.

## Related literature

For preparation of the title compound, see: Gowda *et al.* (2005 ▶). For our study of the effects of substituents on the structures of *N*-(ar­yl)-aryl­sulfonamides, see: Gowda *et al.* (2008 ▶; 2009**a* ▶,b* ▶). For related structures, see: Gelbrich *et al.* (2007 ▶); Perlovich *et al.* (2006 ▶).
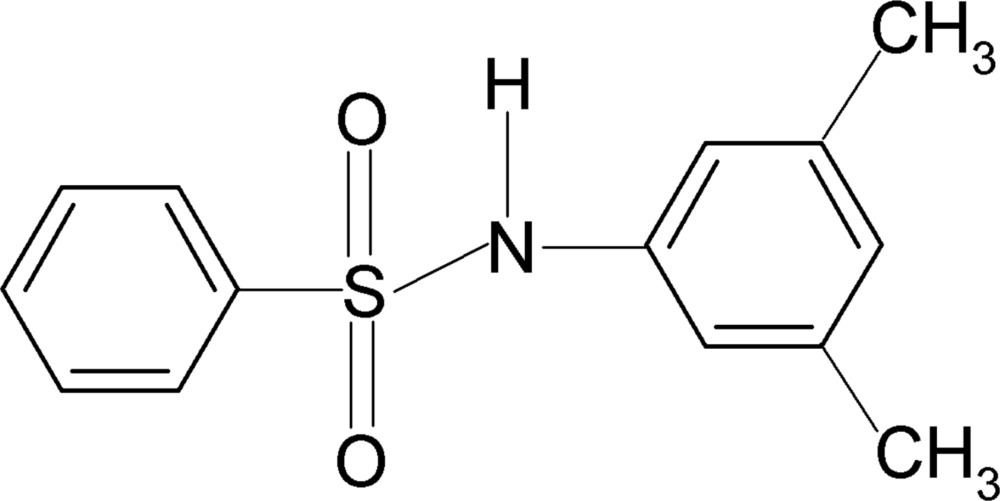


## Experimental

### 

#### Crystal data

C_14_H_15_NO_2_S
*M* *_r_* = 261.33
Monoclinic, 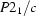

*a* = 11.192 (1) Å
*b* = 7.3543 (7) Å
*c* = 16.672 (2) Å
β = 101.62 (1)°
*V* = 1344.1 (2) Å^3^
*Z* = 4
Mo *K*α radiation
μ = 0.23 mm^−1^
*T* = 299 K
0.48 × 0.40 × 0.18 mm

#### Data collection

Oxford Diffraction Xcalibur diffractometer with a Sapphire CCD detector
Absorption correction: multi-scan (*CrysAlis RED*; Oxford Diffraction, 2009 ▶) *T*_min_ = 0.896, *T*_max_ = 0.959
5063 measured reflections
2742 independent reflections
2187 reflections with *I* > 2σ(*I*)
*R*_int_ = 0.015

#### Refinement

*R*[*F* ^2^ > 2σ(*F* ^2^)] = 0.038
*wR*(*F* ^2^) = 0.111
*S* = 1.05
2742 reflections
169 parameters
H atoms treated by a mixture of independent and constrained refinement
Δρ_max_ = 0.31 e Å^−3^
Δρ_min_ = −0.31 e Å^−3^

### 

Data collection: *CrysAlis CCD* (Oxford Diffraction, 2009 ▶); cell refinement: *CrysAlis RED* (Oxford Diffraction, 2009 ▶); data reduction: *CrysAlis RED*; program(s) used to solve structure: *SHELXS97* (Sheldrick, 2008 ▶); program(s) used to refine structure: *SHELXL97* (Sheldrick, 2008 ▶); molecular graphics: *PLATON* (Spek, 2009 ▶); software used to prepare material for publication: *SHELXL97*.

## Supplementary Material

Crystal structure: contains datablocks I, global. DOI: 10.1107/S1600536809050089/bx2251sup1.cif

Structure factors: contains datablocks I. DOI: 10.1107/S1600536809050089/bx2251Isup2.hkl

Additional supplementary materials:  crystallographic information; 3D view; checkCIF report

## Figures and Tables

**Table 1:** Hydrogen-bond geometry (Å, °)

*D*—H⋯*A*	*D*—H	H⋯*A*	*D*⋯*A*	*D*—H⋯*A*
N1—H1*N*⋯O1^i^	0.81 (2)	2.14 (2)	2.942 (2)	176 (2)

Symmetry code: (i) 
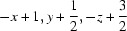
.

**Fig. 1.**
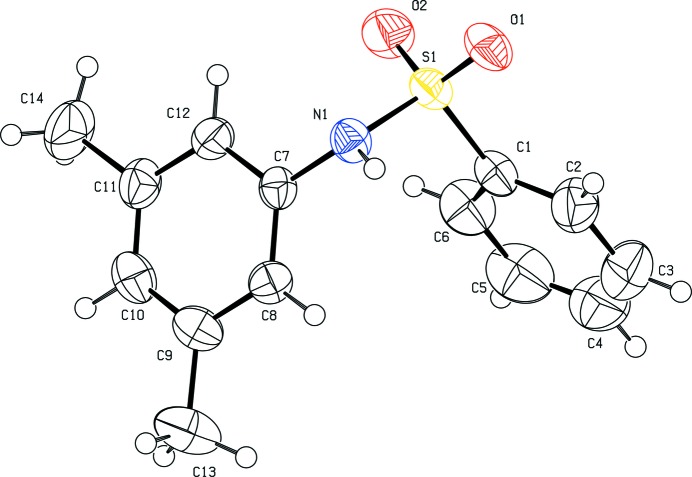
Molecular structure of (I), showing the atom labelling scheme and displacement ellipsoids are drawn at the 50% probability level.

**Fig. 2.**
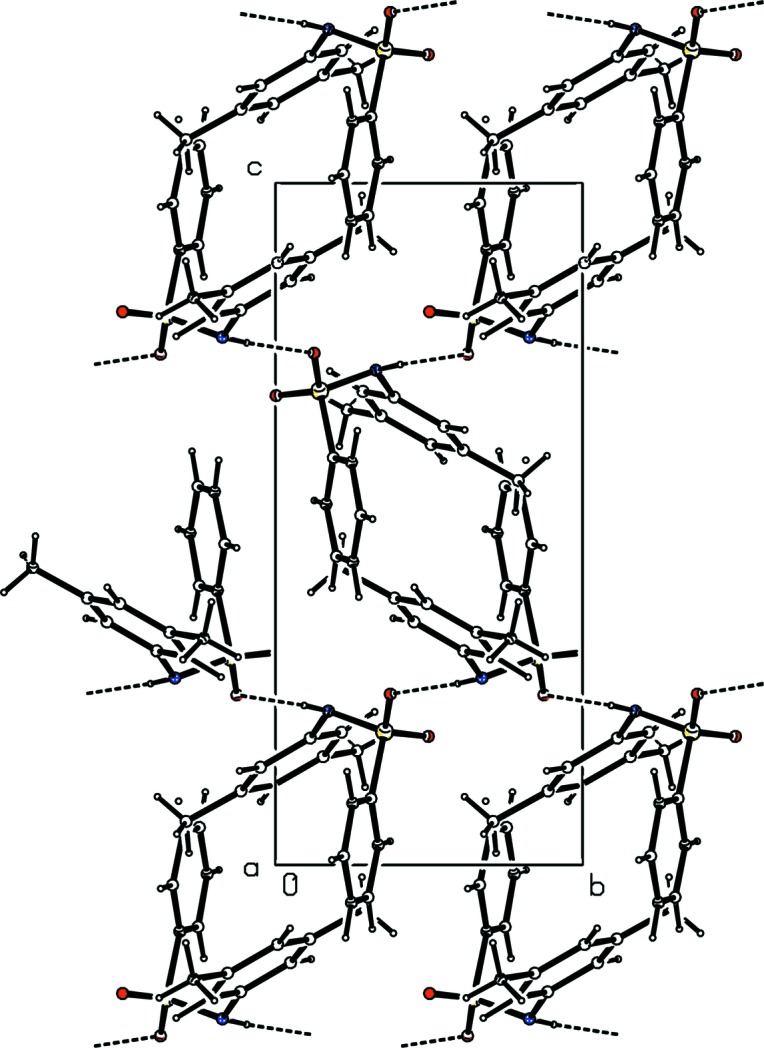
Molecular packing of (I) with hydrogen bonding shown as dashed lines.

## supplementary crystallographic information

### Comment

As part of a study of substituent effects on the structures of *N*-(aryl)-arylsulfonamides (Gowda *et al.*, 2008; 2009*a,b*), in the present work, the structure of *N*-(3,5-dimethylphenyl)benzenesulfonamide (I) has been determined (Fig. 1). The molecule is bent at the *S* atom with the C1—SO_2_—NH—C7 torsion angle of 67.9 (2)°, compared to the values of 71.0 (2)° in *N*-(2,3-dimethylphenyl)benzenesulfonamide (II)(Gowda *et al.*, 2009*a*), 62.7 (2)° in *N*-(2,5-dimethylphenyl)benzenesulfonamide (III) (Gowda *et al.*, 2009*b*) and -78.7 (2)° in *N*-(2,6-dimethylphenyl)benzenesulfonamide (IV)(Gowda *et al.*, 2008). The two benzene rings in (I) are tilted relative to each other by 54.6 (1)°, compared to the values of 64.8 (1)° in (II), 40.4 (1)° in (III) and 44.9 (1)° in (IV). The other bond parameters in (I) are similar to those observed in (II), (III), (IV) and other aryl sulfonamides (Perlovich *et al.*, 2006; Gelbrich *et al.*, 2007). The crystal packing of molecules in (I) *via* N—H···O(S) hydrogen bonds (Table 1) is shown in Fig.2.

### Experimental

The solution of benzene (10 cc) in chloroform (40 cc) was treated dropwise with chlorosulfonic acid (25 cc) at 0 ° C. After the initial evolution of hydrogen chloride subsided, the reaction mixture was brought to room temperature and poured into crushed ice in a beaker. The chloroform layer was separated, washed with cold water and allowed to evaporate slowly. The residual benzenesulfonylchloride was treated with 3,5-dimethylaniline in the stoichiometric ratio and boiled for ten minutes. The reaction mixture was then cooled to room temperature and added to ice cold water (100 cc). The resultant solid *N*-(3,5-dimethylphenyl)benzenesulfonamide was filtered under suction and washed thoroughly with cold water. It was then recrystallized to constant melting point from dilute ethanol. The purity of the compound was checked and characterized by recording its infrared and NMR spectra (Gowda *et al.*, 2005). The single crystals used in X-ray diffraction studies were grown in ethanolic solution by a slow evaporation at room temperature.

### Refinement

The H atom of the NH was located in difference map and its positional parameters were refined freely [N—H = 0.81 (2) Å]. The other H atoms were positioned with idealized geometry using a riding model [C—H = 0.93—0.96 Å]. All H atoms were refined with isotropic displacement parameters (set to 1.2 times of the *U*_eq_ of the parent atom).

### Crystal data

**Table:**

C_14_H_15_NO_2_S	*F*(000) = 552
*M**_r_* = 261.33	*D*_x_ = 1.291 Mg m^−^^3^
Monoclinic, *P*2_1_/*c*	Mo *K*α radiation, λ = 0.71073 Å
Hall symbol: -P 2ybc	Cell parameters from 25 reflections
*a* = 11.192 (1) Å	θ = 2.5–27.8°
*b* = 7.3543 (7) Å	µ = 0.23 mm^−^^1^
*c* = 16.672 (2) Å	*T* = 299 K
β = 101.62 (1)°	Prism, colourless
*V* = 1344.1 (2) Å^3^	0.48 × 0.40 × 0.18 mm
*Z* = 4	

### Data collection

**Table:**

Oxford Diffraction Xcalibur diffractometer with a Sapphire CCD detector	2742 independent reflections
Radiation source: fine-focus sealed tube	2187 reflections with *I* > 2σ(*I*)
graphite	*R*_int_ = 0.015
Rotation method data acquisition using ω and phi scans	θ_max_ = 26.4°, θ_min_ = 2.5°
Absorption correction: multi-scan (*CrysAlis RED*; Oxford Diffraction, 2009)	*h* = −13→13
*T*_min_ = 0.896, *T*_max_ = 0.959	*k* = −6→9
5063 measured reflections	*l* = −9→20

### Refinement

**Table:**

Refinement on *F*^2^	Secondary atom site location: difference Fourier map
Least-squares matrix: full	Hydrogen site location: inferred from neighbouring sites
*R*[*F*^2^ > 2σ(*F*^2^)] = 0.038	H atoms treated by a mixture of independent and constrained refinement
*wR*(*F*^2^) = 0.111	*w* = 1/[σ^2^(*F*_o_^2^) + (0.0575*P*)^2^ + 0.3783*P*] where *P* = (*F*_o_^2^ + 2*F*_c_^2^)/3
*S* = 1.05	(Δ/σ)_max_ = 0.004
2742 reflections	Δρ_max_ = 0.31 e Å^−^^3^
169 parameters	Δρ_min_ = −0.31 e Å^−^^3^
0 restraints	Extinction correction: *SHELXL97* (Sheldrick, 2008), Fc^*^=kFc[1+0.001xFc^2^λ^3^/sin(2θ)]^-1/4^
Primary atom site location: structure-invariant direct methods	Extinction coefficient: 0.0115 (18)

### Special details

**Table:**

Experimental. CrysAlis RED (Oxford Diffraction, 2009) Empirical absorption correction using spherical harmonics, implemented in SCALE3 ABSPACK scaling algorithm.
Geometry. All e.s.d.'s (except the e.s.d. in the dihedral angle between two l.s. planes) are estimated using the full covariance matrix. The cell e.s.d.'s are taken into account individually in the estimation of e.s.d.'s in distances, angles and torsion angles; correlations between e.s.d.'s in cell parameters are only used when they are defined by crystal symmetry. An approximate (isotropic) treatment of cell e.s.d.'s is used for estimating e.s.d.'s involving l.s. planes.
Refinement. Refinement of *F*^2^ against ALL reflections. The weighted *R*-factor *wR* and goodness of fit *S* are based on *F*^2^, conventional *R*-factors *R* are based on *F*, with *F* set to zero for negative *F*^2^. The threshold expression of *F*^2^ > σ(*F*^2^) is used only for calculating *R*-factors(gt) *etc*. and is not relevant to the choice of reflections for refinement. *R*-factors based on *F*^2^ are statistically about twice as large as those based on *F*, and *R*- factors based on ALL data will be even larger.

### Fractional atomic coordinates and isotropic or equivalent isotropic displacement parameters (Å^2^)

**Table:**

	*x*	*y*	*z*	*U*_iso_*/*U*_eq_	
C1	0.41245 (16)	0.1889 (3)	0.59746 (11)	0.0434 (4)	
C2	0.52681 (19)	0.2478 (3)	0.59053 (14)	0.0618 (6)	
H2	0.5873	0.2668	0.6369	0.074*	
C3	0.5495 (3)	0.2780 (4)	0.51321 (17)	0.0816 (8)	
H3	0.6266	0.3161	0.5074	0.098*	
C4	0.4597 (3)	0.2524 (4)	0.44490 (15)	0.0790 (8)	
H4	0.4764	0.2716	0.3931	0.095*	
C5	0.3458 (3)	0.1986 (4)	0.45274 (14)	0.0805 (8)	
H5	0.2846	0.1846	0.4063	0.097*	
C6	0.3209 (2)	0.1650 (3)	0.52926 (13)	0.0620 (6)	
H6	0.2438	0.1268	0.5347	0.074*	
C7	0.20322 (14)	0.3865 (2)	0.68610 (10)	0.0354 (4)	
C8	0.18940 (16)	0.5491 (2)	0.64340 (10)	0.0410 (4)	
H8	0.2578	0.6162	0.6381	0.049*	
C9	0.07428 (18)	0.6121 (3)	0.60856 (12)	0.0479 (5)	
C10	−0.02579 (17)	0.5069 (3)	0.61670 (12)	0.0514 (5)	
H10	−0.1035	0.5477	0.5929	0.062*	
C11	−0.01416 (16)	0.3444 (3)	0.65873 (12)	0.0489 (5)	
C12	0.10215 (16)	0.2850 (3)	0.69465 (11)	0.0430 (4)	
H12	0.1122	0.1772	0.7244	0.052*	
C13	0.0577 (2)	0.7909 (3)	0.56388 (16)	0.0733 (7)	
H13A	0.1323	0.8239	0.5476	0.088*	
H13B	0.0364	0.8830	0.5993	0.088*	
H13C	−0.0063	0.7799	0.5162	0.088*	
C14	−0.12452 (19)	0.2338 (4)	0.66755 (16)	0.0755 (7)	
H14A	−0.1717	0.2058	0.6142	0.091*	
H14B	−0.1735	0.3023	0.6978	0.091*	
H14C	−0.0985	0.1229	0.6962	0.091*	
N1	0.32271 (13)	0.3272 (2)	0.72571 (9)	0.0395 (4)	
H1N	0.3739 (18)	0.406 (3)	0.7338 (12)	0.047*	
O1	0.49861 (12)	0.12569 (19)	0.75071 (8)	0.0526 (4)	
O2	0.29563 (13)	0.00239 (19)	0.68847 (10)	0.0590 (4)	
S1	0.38310 (4)	0.14444 (6)	0.69537 (3)	0.04071 (17)	

### Atomic displacement parameters (Å^2^)

**Table:**

	*U*^11^	*U*^22^	*U*^33^	*U*^12^	*U*^13^	*U*^23^
C1	0.0437 (10)	0.0417 (10)	0.0431 (9)	0.0098 (8)	0.0043 (8)	−0.0040 (8)
C2	0.0500 (12)	0.0781 (15)	0.0577 (13)	0.0046 (11)	0.0119 (10)	0.0035 (11)
C3	0.0782 (18)	0.100 (2)	0.0758 (17)	0.0074 (16)	0.0366 (14)	0.0110 (16)
C4	0.110 (2)	0.0806 (18)	0.0512 (14)	0.0199 (16)	0.0283 (15)	0.0035 (12)
C5	0.104 (2)	0.0826 (18)	0.0466 (13)	0.0137 (16)	−0.0035 (13)	−0.0090 (12)
C6	0.0616 (14)	0.0681 (14)	0.0509 (12)	0.0016 (11)	−0.0019 (10)	−0.0081 (10)
C7	0.0323 (8)	0.0407 (9)	0.0330 (8)	0.0014 (7)	0.0063 (6)	−0.0033 (7)
C8	0.0411 (9)	0.0395 (9)	0.0419 (9)	−0.0038 (8)	0.0074 (7)	−0.0010 (7)
C9	0.0507 (11)	0.0427 (10)	0.0458 (10)	0.0040 (8)	−0.0008 (8)	−0.0007 (8)
C10	0.0365 (10)	0.0596 (12)	0.0539 (11)	0.0079 (9)	−0.0012 (8)	−0.0014 (10)
C11	0.0354 (9)	0.0621 (12)	0.0495 (11)	−0.0036 (8)	0.0093 (8)	0.0006 (9)
C12	0.0393 (9)	0.0463 (10)	0.0441 (10)	−0.0018 (8)	0.0101 (7)	0.0058 (8)
C13	0.0773 (16)	0.0504 (13)	0.0804 (17)	0.0035 (12)	−0.0119 (13)	0.0119 (12)
C14	0.0411 (12)	0.100 (2)	0.0858 (17)	−0.0124 (12)	0.0142 (11)	0.0160 (15)
N1	0.0326 (8)	0.0392 (8)	0.0450 (8)	−0.0012 (6)	0.0038 (6)	−0.0030 (6)
O1	0.0436 (7)	0.0577 (8)	0.0510 (7)	0.0142 (6)	−0.0032 (6)	0.0062 (6)
O2	0.0556 (8)	0.0410 (7)	0.0798 (10)	−0.0062 (6)	0.0123 (7)	−0.0002 (7)
S1	0.0367 (3)	0.0373 (3)	0.0457 (3)	0.00433 (18)	0.00246 (18)	0.00225 (18)

### Geometric parameters (Å, °)

**Table:**

C1—C2	1.378 (3)	C9—C10	1.390 (3)
C1—C6	1.380 (3)	C9—C13	1.505 (3)
C1—S1	1.7594 (19)	C10—C11	1.378 (3)
C2—C3	1.381 (3)	C10—H10	0.9300
C2—H2	0.9300	C11—C12	1.389 (2)
C3—C4	1.372 (4)	C11—C14	1.511 (3)
C3—H3	0.9300	C12—H12	0.9300
C4—C5	1.366 (4)	C13—H13A	0.9600
C4—H4	0.9300	C13—H13B	0.9600
C5—C6	1.382 (3)	C13—H13C	0.9600
C5—H5	0.9300	C14—H14A	0.9600
C6—H6	0.9300	C14—H14B	0.9600
C7—C8	1.384 (2)	C14—H14C	0.9600
C7—C12	1.386 (2)	N1—S1	1.6302 (15)
C7—N1	1.435 (2)	N1—H1N	0.81 (2)
C8—C9	1.382 (2)	O1—S1	1.4356 (13)
C8—H8	0.9300	O2—S1	1.4204 (14)
			
C2—C1—C6	121.3 (2)	C9—C10—H10	118.8
C2—C1—S1	119.10 (15)	C10—C11—C12	118.44 (17)
C6—C1—S1	119.64 (16)	C10—C11—C14	121.40 (19)
C1—C2—C3	118.5 (2)	C12—C11—C14	120.15 (19)
C1—C2—H2	120.7	C7—C12—C11	119.97 (17)
C3—C2—H2	120.7	C7—C12—H12	120.0
C4—C3—C2	120.7 (2)	C11—C12—H12	120.0
C4—C3—H3	119.6	C9—C13—H13A	109.5
C2—C3—H3	119.6	C9—C13—H13B	109.5
C5—C4—C3	120.2 (2)	H13A—C13—H13B	109.5
C5—C4—H4	119.9	C9—C13—H13C	109.5
C3—C4—H4	119.9	H13A—C13—H13C	109.5
C4—C5—C6	120.4 (2)	H13B—C13—H13C	109.5
C4—C5—H5	119.8	C11—C14—H14A	109.5
C6—C5—H5	119.8	C11—C14—H14B	109.5
C1—C6—C5	118.9 (2)	H14A—C14—H14B	109.5
C1—C6—H6	120.5	C11—C14—H14C	109.5
C5—C6—H6	120.5	H14A—C14—H14C	109.5
C8—C7—C12	120.60 (16)	H14B—C14—H14C	109.5
C8—C7—N1	119.75 (15)	C7—N1—S1	120.92 (12)
C12—C7—N1	119.56 (16)	C7—N1—H1N	115.0 (15)
C9—C8—C7	120.24 (16)	S1—N1—H1N	108.7 (15)
C9—C8—H8	119.9	O2—S1—O1	119.88 (9)
C7—C8—H8	119.9	O2—S1—N1	108.03 (8)
C8—C9—C10	118.28 (18)	O1—S1—N1	104.82 (8)
C8—C9—C13	120.85 (19)	O2—S1—C1	108.42 (9)
C10—C9—C13	120.87 (18)	O1—S1—C1	107.56 (9)
C11—C10—C9	122.46 (17)	N1—S1—C1	107.53 (8)
C11—C10—H10	118.8		
			
C6—C1—C2—C3	−1.8 (3)	C8—C7—C12—C11	1.4 (3)
S1—C1—C2—C3	178.45 (19)	N1—C7—C12—C11	177.90 (16)
C1—C2—C3—C4	0.9 (4)	C10—C11—C12—C7	−1.5 (3)
C2—C3—C4—C5	0.8 (4)	C14—C11—C12—C7	179.70 (19)
C3—C4—C5—C6	−1.7 (4)	C8—C7—N1—S1	−114.44 (16)
C2—C1—C6—C5	0.9 (3)	C12—C7—N1—S1	69.0 (2)
S1—C1—C6—C5	−179.33 (19)	C7—N1—S1—O2	−48.89 (16)
C4—C5—C6—C1	0.9 (4)	C7—N1—S1—O1	−177.81 (13)
C12—C7—C8—C9	−0.1 (3)	C7—N1—S1—C1	67.93 (15)
N1—C7—C8—C9	−176.67 (16)	C2—C1—S1—O2	−148.57 (17)
C7—C8—C9—C10	−0.9 (3)	C6—C1—S1—O2	31.65 (19)
C7—C8—C9—C13	178.38 (19)	C2—C1—S1—O1	−17.56 (19)
C8—C9—C10—C11	0.7 (3)	C6—C1—S1—O1	162.66 (16)
C13—C9—C10—C11	−178.5 (2)	C2—C1—S1—N1	94.86 (17)
C9—C10—C11—C12	0.5 (3)	C6—C1—S1—N1	−84.93 (18)
C9—C10—C11—C14	179.3 (2)		

### Hydrogen-bond geometry (Å, °)

**Table:**

*D*—H···*A*	*D*—H	H···*A*	*D*···*A*	*D*—H···*A*
N1—H1N···O1^i^	0.81 (2)	2.14 (2)	2.942 (2)	176 (2)

Symmetry codes: (i) −*x*+1, *y*+1/2, −*z*+3/2.
